# Childhood Stroke and Congenital Heart Disease

**DOI:** 10.15844/pedneurbriefs-29-3-1

**Published:** 2015-03

**Authors:** Laurence Ducharme-Crevier, Mark S. Wainwright

**Affiliations:** 1 Ruth D. & Ken M. Davee Pediatric Neurocritical Care Program, Ann & Robert H. Lurie Children's Hospital of Chicago, Chicago, IL; 2 Departments of Pediatrics and Neurology, Northwestern University Feinberg School of Medicine, Chicago, IL

**Keywords:** Epidemiology, Stroke, Pediatrics, Congenital Heart Disease

## Abstract

Investigators from the University of California performed a case-control study of the association of stroke with congenital heart disease (CHD) within a population of 2.5 million children in Northern California.

Investigators from the University of California performed a case-control study of the association of stroke with congenital heart disease (CHD) within a population of 2.5 million children in Northern California [1]. Cases of ischemic or hemorrhagic stroke were identified from review of medical records using a combination clinical symptoms and neuroimaging as they have previously described [2]. Among the 412 cases of stroke, 15 (4%) had a history of CHD. In contrast, 7 of the 1236 stroke-free controls had a history of CHD (prevalence 0.6%). Both cyanotic and acyanotic CHD were associated with stroke. Children with stroke and CHD presented at a younger age than non-CHD children. Children with CHD and previous cardiac surgery had a 31-fold increased stroke risk compared with healthy children. Importantly, the majority of the non-neonatal cases of stroke with CHD occurred after hospital discharge. Almost half the children with stroke, CDH and previous cardiac procedure presented within 5 years of their most recent cardiac surgery. The risk for stroke persists long after cardiac surgery as one patient presented with stroke 18 years after the last intervention. Both groups were similar in regard to therapy strategy, as 55% of all stroke patients were treated with an antithrombotic agent. A high proportion of patients had a neurological deficit at the time of hospital discharge. [1]

COMMENTARY. The importance of CHD as a risk factor for ischemic and hemorrhagic pediatric stroke is well established [3]. This new study reports a lower proportion of ischemic stroke attributed to CHD, probably in the context of a community-based study rather than referral-based. Of note, both cyanotic and non-cyanotic lesions were associated with stroke in children. A key finding of this study are the data that suggest a long-term increase in this risk for stroke after surgery for CHD. While the study is limited by the small number of cases of stroke and controls with CHD these results identify a long-term risk for stroke in children with CHD. There is a growing population of adult survivors of CHD and recognition of the cognitive issues faced by these patients [4]. The results of this study underscore the importance of anticipatory guidance for parents and caregivers after hospital discharge. The findings highlight an important gap in our understanding of the approach to neuroprotection in the post-operative period and stroke prophylaxis during long-term recovery for this expanding population.
